# Supplementary material for: Implication between Genetic Variants from APOA5 and ZPR1 and NAFLD Severity in Patients with Hypertriglyceridemia
Source: Nutrients. 2021 Feb 8;13(2):552. doi: 10.3390/nu13020552 (PMC7914661; doi:10.3390/nu13020552)
Supplement: Supplementary file 1 [file nutrients-13-00552-s001.pdf]

SUPLEMENTARY MATERIAL

Table S1 (Supplementary material). Genotype frequencies of the variables analyzed of the genes BAZ1B, GCKR, LPL and TRIB.

		**Non/mild**	**Moderate/Severe**		
**Gene**	**SNP**	**N**	**N**	**X^2**	**p-value**
**BAZ1B**	**Rs7811265**			**NA**	
	CC	5 (5.6)	3 (3.2)		
	CT	14 (15.7)	15 (15.8)		
	TT	70 (78.7)	77 (81.1)		
Dominant model	CT+TT	84 (94.4)	92 (96.8)	NA	0.486*
Recessive model	TT	70 (78.7)	77 (81.1)	0.165	0.685
**GCKR**	**Rs1260326**			1.568	0.457
	CC	12 (13.5)	17 (17.7)		
	CT	50 (56.2)	57 (59.4)		
	TT	27 (30.3)	22 (22.9)		
Dominant model	CT+TT	84 (94.4)	92 (96.8)	0.624	0.430
Recessive model	TT	27 (30.3)	22 (22.9)	1.306	0.253
**LPL**	**Rs12678919**			NA	
	GG	1 (1.1)	0 (0)		
	GA	9 (10.2)	12 (12.6)		
	AA	78 (88.6)	83 (87.4)		
Dominant model	GA+AA	87 (98.9)	95 (100)	NA	0.481*
Recessive model	AA	78 (88.6)	83 (87.4)	0.069	0.792
**TRIB**	**Rs2954029**			0.615	0.735
	TT	9 (10.1)	8 (8.3)		
	TA	45 (50.6)	45 (46.9)		
	AA	35 (39.3)	43 (44.8)		
Dominant model	TA+AA	80 (89.9)	88 (91.7)	0.175	0.676
Recessive model	AA	35 (39.3)	43 (44.8)	0.566	0.452

Data are expressed as absolute numbers and percentages by NAFLD severity group. * p-value of Fisher exact test. In the dominant model homozygotes and heterozygotes are compared with non-mutant (CT+TT vs. CC / GA+AA vs GG / TA+AA vs. TT), and in the recessive model homozygotes were compared with heterozygotes and non-mutant (TT vs. CT+CC / AA vs. GA+GG / AA vs. TA+TT). Abbreviations: SNP: single nucleotide polymorphism; NA: not applicable.

Table S2 (Supplementary material). Association of genetic variants with metabolic traits.

BAZ1B (rs 7811265)	Wild type CC	Heterozygous CT	Homozygous TT	*P*
**TC** mmol/L	7.20 (1.73)	6.67 (1.94)	6.70 (1.77)	0.660
**TG** mmol/L*	14.1 (3.6-19.2)	4.3 (2.6-8.7)	5.8 (4-11.1)	0.025
**Non-HDL** mmol/L	6.45 (1.73)	5.58 (1.95)	5.73 (1.78)	0.353
**HDL-c** mmol/L	0.74 (0.26)	1.03 (0.32)	0.97 (0.31)	0.021
**Glucose** mmol/L*	6.5 (5.2-7.7)	5.3 (5-6)	5.6 (5.1-6.4)	0.124
**Insulin** pmol/L*	142 (109.6-309)	95.6 (84.9-134)	116.5 (75.6-163.5)	0.196
**HOMA-IR***	6.3 (4.4-14)	3.6 (2.99-4.9)	4.2 (2.6-6.3)	0.105
**AST** µkat/L*	0.32 (0.26-065)	0.46 (0.41-0.51)	0.41 (0.33-0.52)	0.054
**ALT** µkat/L*	0.33 (0.23-0.79)	0.58 (0.45-0.77)	0.47 (0.35-0.78)	0.120
**CRP—hs** mg/L*	1.1 (0.9-5.5)	0.8 (0.6-2.3)	1 (0.6-3.3)	0.492
**Lp(a) nmol/L***	36.4(7-88.4)	28.2(7-182.6)	42.7(8-115)	0.800
**GCKR (rs 1260326)**	**Wild type CC**	**Heterozygous CT**	**Homozygous TT**	***P***
**TC** mmol/L	6.50 (1.85)	6.71 (1.83)	6.87 (1.65)	0.063
**TG** mmol/L*	4.8 (3.1-8.1)	5.6 (3.7-9.8)	7.3 (4-12)	0.110
**Non-HDL** mmol/L	5.57 (1.82)	5.70 (1.85)	5.93 (1.68)	0.512
**HDL-c** mmol/L	0.94 (0.25)	0.99 (0.34)	0.95 (0.30)	0.423
**Glucose** mmol/L*	6.1 (5.2-6.8)	5.7 (5.2-6.4)	5.3 (4.9-5.9)	0.019
**Insulin** pmol/L*	109 (78.9-143)	124.5 (76.9-170.3)	96.5 (74.7-134.5)	0.334
**HOMA-IR***	4.4 (2.9-5.9)	4.5 (2.9-6.5)	3.2 (2.6-5.2)	0.140
**AST** µkat/L*	0.39 (0.33-0.59)	0.42 (0.35-0.50)	0.44- (0.32-0.5)	0.992
**ALT** µkat/L*	0.46 (0.36-0.84)	0.5 (0.35-0.77)	0.51 (0.35-0.77)	0.968
**CRP—hs** mg/L*	1.1 (0.7-3.1)	0.9 (0.6-2.4)	1.3 (0.7-3.5)	0.200
**Lp(a) nmol/L***	21.3(7-84)	35.8(7-101)	59.5(9.6-190)	0.142
**LPL (rs 12678919)**	**Wild type GG**	**Heterozygous AG**	**Homozygous AA**	***P***
**TC** mmol/L		6.85 (2.18)	6.69 (1.74)	0.894
**TG** mmol/L*		5.3 (3.3-16.4)	5.8 (3.8-10.3)	0.537
**Non-HDL** mmol/L		5.85 (2.23)	5.73 (1.75)	0.918
**HDL-c** mmol/L		0.99 (0.44)	0.97 (0.30)	0.733
**Glucose** mmol/L*		5.8 (5.1-6.6)	5.5 (5.1-6.3)	0.428
**Insulin** pmol/L*		94.1 (51.5-142)	114 (77-158)	0.553
**HOMA-IR***		3.9 (2.1-6.1)	4.2 (2.7-6.3)	0.642
**AST** µkat/L*		0.41 (0.33-49)	0.42 (0.33-0.53)	0.303
**ALT** µkat/L*		0.40 (0.33-0.65)	0.5 (0.36-0.77)	0.180
**CRP—hs** mg/L*		1.1 (0.8-3.8)	1 (0.6-2.6)	0.353
**Lp(a) nmol/L***		14(7-90)	39.2(7.2-154)	0.375
**TRIB (rs 2954029)**	**Wild type TT**	**Heterozygous TA**	**Homozygous AA**	***P***
**TC** mmol/L	6.27 (1.65)	6.56 (1.74)	6.98 (1.84)	0.062
**TG** mmol/L*	7.36 (3.87-13.19)	5.41 (3.23-9)	6.02 (4.03-13.12)	0.168
**Non-HDL** mmol/L	5.27 (1.57)	5.59 (1.78)	6.00 (1.86)	0.068
**HDL-c** mmol/L	0.99 (0.39)	0.97 (0.30)	0.97 (0.31)	0.903
**Glucose** mmol/L*	5.6 (5.2-6.8)	5.5 (5.1-6.4)	5.6 (4.9-6.2)	0.536
**Insulin** pmol/L*	94.8 (79.8-124)	115 (78.5-156.5)	124 (75.4-165)	0.628
**HOMA-IR***	3.3 (2.69-4.98)	4.11 (2.88-5.86)	4.41 (2.62-6.41)	0.675
**AST** µkat/L*	0.37 (0.33-0.5)	0.4 (0.33-0.48)	0.45 (0.33-0.6)	0.121
**ALT** µkat/L*	0.48 (0.34-0.63)	0.47 (0.35-0.68)	0.56 (0.37-0.99)	0.205

**CRP—hs** mg/L*	1.3 (0.8-5.3)	1 (0.6-2.6)	0.9 (0.5-2.8)	0.315
**Lp(a) nmol/L***	41.6(7.8-94.4)	47(7-150.7)	31.9(8.-152.5)	0.862

Data are expressed as percentage (analysed by Chi-square) or mean (+/-SD) for normal quantitative variables (analysed by ANOVA test) or median and interquartile interval for non-normal quantitative variables (analysed by Kruskall-Wallis test). Abbreviations: TC (total cholesterol concentration), Tg (triglycerides concentration), non-HDL (total cholesterol except HDL), HOMA-IR (Homeostasis Model Assessment), AST (aspartate amino transferase), ALT (alanine amino transferase), CRP-hs (C-reactive protein high sensitivity) , Lp(a): lipoprotein a. *Non-normal quantitative variables.
